# Significance and *In Vivo* Detection of Iron-Laden Microglia in White Matter Multiple Sclerosis Lesions

**DOI:** 10.3389/fimmu.2018.00255

**Published:** 2018-02-19

**Authors:** Kelly M. Gillen, Mayyan Mubarak, Thanh D. Nguyen, David Pitt

**Affiliations:** ^1^Department of Radiology, Weill Cornell Medicine, New York, NY, United States; ^2^Department of Neurology, Yale School of Medicine, New Haven, CT, United States

**Keywords:** multiple sclerosis, microglia, magnetic resonance imaging, quantitative susceptibility mapping, myelin, iron

## Abstract

Microglia are resident immune cells that fulfill protective and homeostatic functions in the central nervous system (CNS) but may also promote neurotoxicity in the aged brain and in chronic disease. In multiple sclerosis (MS), an autoimmune demyelinating disease of the CNS, microglia and macrophages contribute to the development of white matter lesions through myelin phagocytosis, and possibly to disease progression through diffuse activation throughout myelinated white matter. In this review, we discuss an additional compartment of myeloid cell activation in MS, i.e., the rim and normal adjacent white matter of chronic active lesions. In chronic active lesions, microglia and macrophages may contain high amounts of iron, express markers of proinflammatory polarization, are activated for an extended period of time (years), and drive chronic tissue damage. Iron-positive myeloid cells can be visualized and quantified with quantitative susceptibility mapping (QSM), a magnetic resonance imaging technique. Thus, QSM has potential as an *in vivo* biomarker for chronic inflammatory activity in established white matter MS lesions. Reducing chronic inflammation associated with iron accumulation using existing or novel MS therapies may impact disease severity and progression.

## Introduction to Microglia

Microglia are resident immune cells of the central nervous system (CNS) responsible for homeostatic functions, including neurogenesis and clearance of cellular debris, and for responding to injury and infection (1–3). In a resting state, microglia have a ramified appearance with thin processes that survey the surrounding microenvironment (4–6). Following activation, microglia and macrophages can adopt a spectrum of phenotypes composed of pro-inflammatory (M1) and anti-inflammatory (M2) functions (7–11). The classically activated M1 phenotype is characterized by expression of pro-inflammatory cytokines (e.g., IL-1β, TNF-α), and induction of nitric-oxide synthase (6, 12, 13), while the M2 phenotype is characterized by secretion of anti-inflammatory cytokines (e.g., IL-10), and neurotrophic and angiogenic factors (6, 12, 13). Changes in activation status and cell signaling induce morphological changes, motility, and phagocytosis (14). Even though microglia and macrophages express similar cell surface markers and can be morphologically indistinguishable (13, 14), they originate from distinct progenitors: macrophages are monocyte-derived, while microglia arise from differentiated yolk sac erythromyeloid precursors (15–17). Macrophages have been widely studied *in vivo* and *in vitro*; however, the functions of microglia are still not well defined, including their roles in inflammatory diseases such as multiple sclerosis (MS).

## MS Lesion Pathology

Multiple sclerosis is a chronic inflammatory disease of the CNS, characterized by focal demyelination, that is caused by an autoimmune response to self-antigens (18). MS is the most common cause of non-traumatic neurological disability in young adults, affecting more than 2.3 million people worldwide. The disease usually starts with episodes of neurological dysfunction that remit spontaneously, a course that is termed relapsing remitting MS (RRMS). One to two decades into RRMS, most MS patients enter a secondary progressive phase, where relapses are replaced by slow, irreversible progression of neurological disability (19). Significant strides have been made in understanding the pathophysiology of relapses; however, progression remains largely unexplained. Multiple lines of evidence suggest that progressive MS is associated with chronic activation of the CNS innate immune system (20–22).

**Figure 1:**
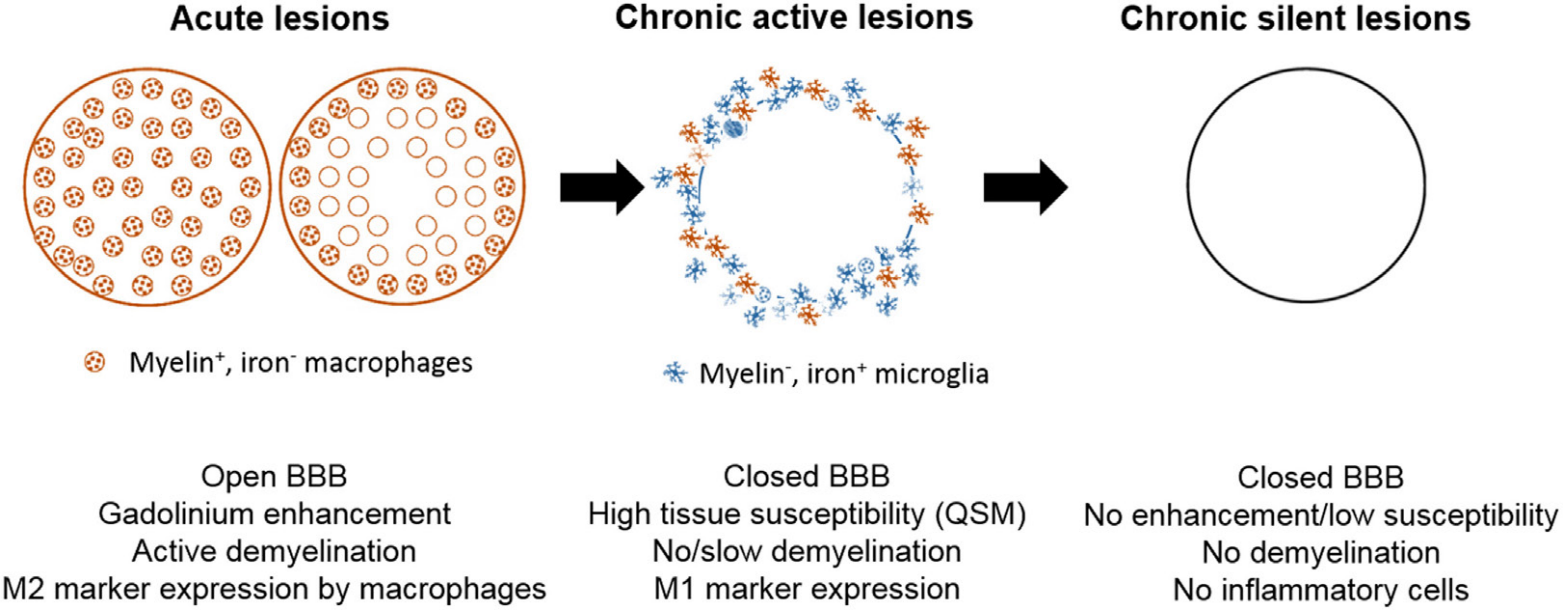
Schematic of white-matter lesion development and its representation with quantitative susceptibility mapping (QSM). Acutely demyelinating lesions are Gd enhancing on T1w imaging and contain M2-activated macrophages. Acute lesions eventually progress to chronic active lesions that may contain iron^+^ microglia/macrophages at the lesion rim and express M1 activation markers. These lesions are typically non-enhancing and appear hyperintense with respect to normal appearing white matter (NAWM) on susceptibility weighted imaging. Chronic silent lesions lack inflammatory cells and their susceptibility is similar to that of nearby NAWM.

Inflammatory demyelinating lesions are a pathological hallmark of RRMS. Acutely demyelinating lesions are characterized by a breach of the blood–brain barrier (BBB), infiltration with leukocytes, and breakdown and phagocytosis of myelin (23). Acute lesions evolve into chronic active lesions, which contain a demyelinated, gliotic lesion center, and activated microglia and macrophages at the lesion edge. Depending on the activation status and phagocytotic activity of myeloid cells at the lesion rim, chronic active lesions may stay dormant or continue to slowly expand (Figure 1) (24). Eventually, chronic active lesions become chronic silent, i.e., they no longer contain inflammatory cells (25). Myelin-laden, foamy macrophages in the center and inner rim of acute lesions express anti-inflammatory cytokines (26), suggesting that myelin phagocytosis induces an anti-inflammatory phenotype, which may contribute to the eventual resolution of inflammation. The M2-inducing properties of myelin uptake have been confirmed in cultured monocyte-derived macrophages and in mouse models of spinal-cord injury (26–29).

A more recent study posits that myelin-containing macrophages in actively demyelinating areas exhibit a mixed phenotype expressing both M1 and M2 markers, including CD40, CD86, CD64, and CD32 (M1), as well as mannose receptor and CD163 (M2) (30). Moreover, at the rim of chronic active lesions, microglia lack expression of M2 markers, suggesting that the M2 component becomes extinguished once the lesion progresses from acutely demyelinating to chronic active (30). M2 markers are also expressed by microglia and macrophages during remyelination (31).

## The Role of Iron in the CNS

A striking feature of chronic active MS lesions is that iron is highly enriched in activated microglia and macrophages at the lesion edge (27), which has implications for their function and *in vivo* detection in MS patients, as discussed below. Iron acts as a cofactor for various enzymatic reactions, and is essential for normal brain function, specifically the synthesis and maintenance of myelin (32, 33). Accordingly, in the CNS, iron is present primarily in oligodendrocytes and myelin (34), where it is stored predominantly in the redox-inactive ferric (Fe^3+^) form within ferritin. Unbound ferrous iron (Fe^2+^) can catalyze production of reactive oxygen species (ROS) through the Fenton reaction (35, 36). This cytotoxic process is minimized through a highly coordinated process that involves specific iron transport, uptake, and storage proteins, including transferrin, transferrin receptor, hepcidin, divalent metal transporter 1, ferroportin, and ferritin (37, 38).

In the normal aging brain, iron levels increase in the cortex, cerebellum, and deep gray matter (39, 40). Accelerated accumulation of iron in the basal ganglia and motor cortex have been demonstrated in several CNS disorders such as Parkinson’s disease (41), Alzheimer’s disease (42), Huntington’s disease (43, 44), amyotrophic lateral sclerosis (45), and MS (46–52). While iron accumulation correlates with disease progression, the pathological processes have not been well delineated. Iron accumulation may be associated with excess ferrous iron and ROS production (53), but it is unknown whether iron accumulation is the cause of tissue damage or occurs secondary to neurodegeneration. Oligodendrocytes, oligodendrocyte progenitors, and neurons are particularly sensitive to ROS, as they are unable, unlike astrocytes, to produce high levels of the free-radical scavenger glutathione (54–56). Glutathione also inhibits an iron-dependent form of programmed cell death, ferroptosis, triggered by iron overload (57–60). Furthermore, high iron induces glutamate release by neurons (61, 62), which potentially leads to excitotixicity in neurons and oligodendrocytes.

In MS, increased iron in deep gray matter has been inferred from T2 hypointensities on magnetic resonance imaging (MRI), although changes in T2 signal can be caused by multiple factors, including inflammation and edema. Clinically, deep gray matter T2 hypointensities correlated with brain atrophy, disability progression, and cognitive impairment (47–50, 63). In a study that used quantitative susceptibility mapping (QSM) rather than T2 signal to map iron content, magnetic susceptibility in basal ganglia of MS patients correlated with decreased performance on basal ganglia–reliant neuropsychological tasks (64). Histologically, iron was present in deep gray matter primarily in oligodendrocytes and myelin fibers, and to a lesser extent, in microglia and astrocytes; In contrast to imaging studies, a statistically significant iron increase in deep gray matter of MS patients compared with controls could not be demonstrated (65).

## Iron is a Marker of Chronic Inflammatory MS Lesions

A second site of iron accumulation in MS is in activated microglia/macrophages at the rim of chronic active lesions (27, 66, 67). Myeloid cells play important roles in iron homeostasis, including iron recycling through erythrophagocytosis (68) and induction of inflammatory hypoferremia (69), which bolsters resistance to infectious diseases. Since microbes depend on iron for growth and survival, its sequestration by macrophages is an important inflammatory response (70, 71). In activated macrophages, accumulation of iron is promoted by IL-6 and IL-1β, which induce the iron regulatory hormone hepcidin (69, 72, 73). Thus, iron accumulation is partially regulated by pro-inflammatory cytokines, consistent with the observation that iron uptake correlates with functional polarization of macrophages/microglia. Classically activated (M1) macrophages *in vitro* take up more iron than M2 or M0 macrophages (27, 74, 75), in keeping with the low iron levels in myelin-laden, M2-polarized macrophages *in vitro* and in acutely demyelinating lesions (26, 27). We have recently confirmed that iron uptake is enhanced in human-induced pluripotent stem cell-derived microglia following M1 polarization (unpublished data). Moreover, iron induces a persistent pro-inflammatory state in macrophages in chronic venous ulcers and spinal-cord injury, thus preventing the physiologic switch from M1 to M2 activation associated with wound healing (74, 75). While the direct effects of iron accumulation on macrophage activation are not completely understood, one proposed mechanism is that high intracellular iron activates nuclear factor-kappa B (NF-κB), leading to expression of NF-κB target genes including pro-inflammatory cytokines (76). In additional preliminary data, we found that iron-positive, chronic active lesions contained substantially more activated microglia/macrophages that expressed iNOS, ferritin, and the phagocytosis marker, MerTK, compared with iron-negative, chronic active lesions.

The source of iron in MS lesions is unknown, but it is tempting to speculate that the destruction of iron-rich myelin and oligodendrocytes during lesion formation leads to iron release into the extracellular space and eventual uptake by myeloid cells. Hametner and colleagues have shown that iron is decreased in oligodendrocytes within NAWM in patients with longstanding disease (67), suggesting a shift of iron from oligodendrocytes to microglia, which may impair the ability of oligodendrocytes to maintain myelin or to remyelinate.

## Detecting Chronic Inflammation in MS Patients

Magnetic resonance imaging is a valuable tool for diagnosing MS and monitoring inflammatory activity in MS patients. Acutely demyelinating lesions can be visualized through gadolinium that accumulates within lesions with temporary breakdown of the BBB (77–79). However, gadolinium enhancement offers only a small window into early inflammatory activity, as the BBB closes within weeks of lesion formation (Figure 1). Gadolinium enhancement in MS lesions is preceded and outlasted by infiltration with immune cells. This has been demonstrated in MS patients with positron emission tomography (PET) imaging studies using radioactive ligands for the 18-kDa translocator protein (TSPO) (80), and with MRI of ultra-small iron-oxide particles that were injected peripherally and detected in activated monocytes/macrophages infiltrating the lesions (81). These imaging results are consistent with histological studies indicating that significant inflammatory activity occurs behind a closed BBB (82).

The therapeutic goal of managing MS patients is to completely suppress CNS inflammation. Thus the inability to detect chronic inflammation in MS with conventional MRI techniques is a significant, unmet need in clinical practice. While TSPO-PET imaging allows for assessment of glial cell activation, PET imaging requires significant infrastructure, is costly, and involves patient exposure to radioactivity, all of which make this method unsuitable for broad use in clinical practice. A solution to the problem of visualizing activated microglia/macrophages in lesions is to exploit their high iron content using novel MRI techniques.

## QSM in MS

Tissue can become magnetized in response to a magnetic field, and the extent of magnetization is known as susceptibility, which arises from unpaired electrons in iron or external sources such as contrast agents. MRI permits visualization of tissue susceptibility through gradient echo (GRE) and phase imaging. These techniques have been used to monitor MS lesions (27, 66, 83, 84), but they cannot quantify or localize iron (85). QSM permits visualization of the sizes and shapes of iron sources, delivers precise estimates of iron concentrations, and distinguishes between susceptibility sources such as iron and calcification (85). QSM maps both ferrous (Fe^2+^) and the substantially more common ferric (Fe^3+^) iron, but cannot distinguish between the two sources. In addition, the presence of lipid macromolecules such as myelin reduces tissue susceptibility, resulting in increased susceptibility in demyelinated lesions. QSM is now widely used by the imaging research community in applications to detect iron, map bone mineralization and monitor drug bio-distribution delivered by magnetic-core nanocarriers (38, 44, 86–103).

**Figure 2:**
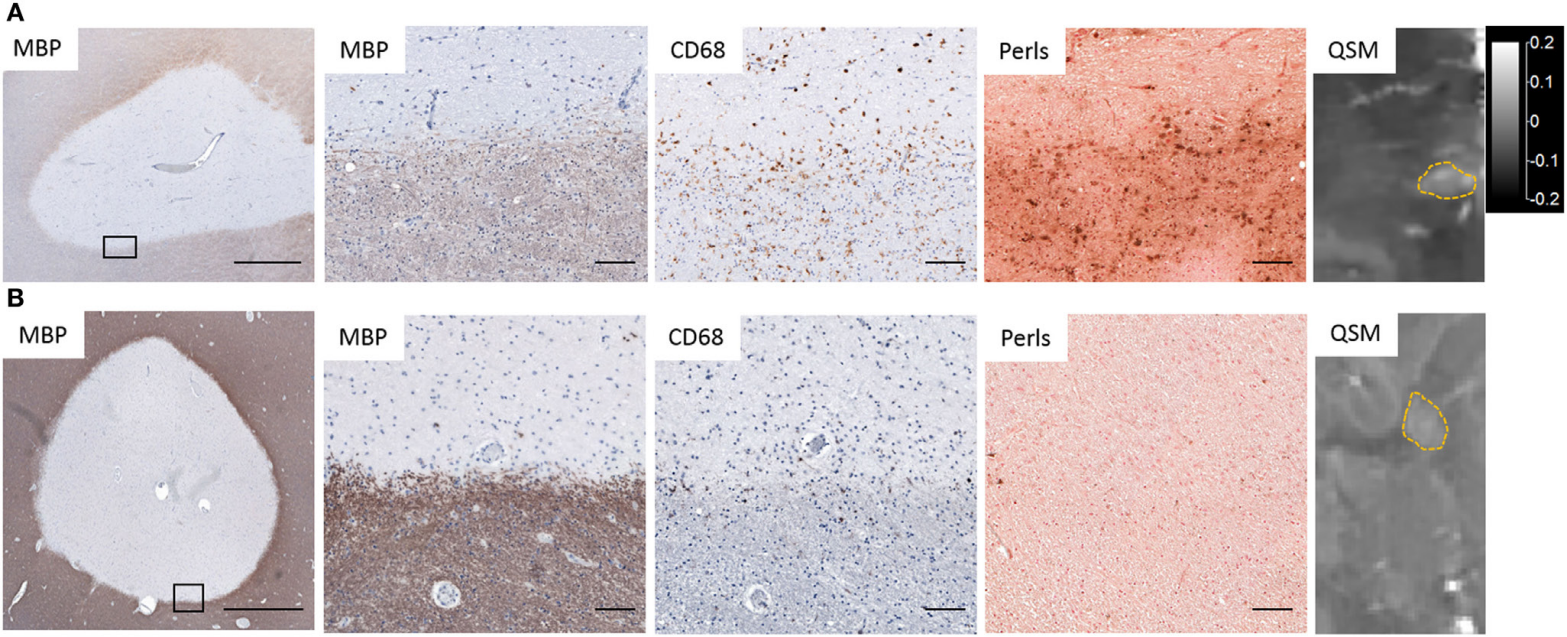
Iron deposition in chronic active lesions corresponding to regions of hyperintensity on quantitative susceptibility mapping (QSM). **(A)** Iron-positive lesions contain CD68^+^ Perls^+^ microglia and macrophages at the lesion rim whose distribution corresponds to hyperintensities on QSM. **(B)** Iron-negative lesions contain few Perls^+^ microglia and have low-tissue susceptibility on QSM. Black rectangles in low magnification images identify the location of higher magnification images. Scale bar in low magnification images = 1,000 µm. Scale bar in high magnification images = 100 µm. QSM scale bar is the same for both images and is in ppm (parts per million).

Several studies, including our own, combined QSM or phase imaging of MS autopsy tissue with histological analysis, and confirmed that high tissue susceptibility at the rims of MS lesions correlated approximately with the distribution of iron and CD68^+^ microglia/macrophages (Figure 2) (24, 27, 66, 83, 104, 105), which contain predominantly ferric iron. In addition, elemental tissue analysis with laser ablation mass spectrometry combined with QSM and IHC of autopsied lesions has established that positive susceptibility values were associated with iron deposition in activated microglia/macrophages (104). In a separate study, X-ray fluorescence imaging and histochemical techniques on autopsied MS brains demonstrated that iron accumulated in microglia/macrophages in chronic lesions (106). These results demonstrate that white matter lesions with high tissue susceptibility at the lesion rim are indicative of iron-positive microglia/macrophages.

An unresolved challenge regarding QSM is the inability to distinguish between the contributions of iron accumulation and myelin loss to lesion susceptibility (107, 108). Both can cause an increase in susceptibility, which generates the need to develop a method to separate the two sources. Birkl et al. addressed the confounding effect of myelin on iron quantification in MS tissue by exploiting the temperature dependency of the susceptibility of paramagnetic iron, which decreases with temperature, while the susceptibility of the diamagnetic myelin remains constant (109). While this technique is well suited for *ex vivo* research, it cannot be applied to patients. In addition, a study of lesions in MS tissue by Wiggermann et al. (108) that determined the sources of lesion contrast on QSM, found a poor correlation between lesional iron content and QSM. While these findings may be explained in part by the low iron content in the examined lesions, their data suggest that the QSM contrast between lesions and the surrounding NAWM may be driven by pathological changes known to be present in NAWM. Therefore, using NAWM as susceptibility reference, as is common in current practice, can lead to an incorrect interpretation of QSM change. A more reliable reference is cerebrospinal fluid, which consists essentially of water and can provide a uniform zero-reference (110).

In the first study that applied QSM to MS, Langkammer et al. demonstrated in patients with established MS or with clinically isolated syndrome, an isolated MS-like neurological episode, that QSM is more sensitive than R2* in the detection of tissue changes in the basal ganglia (107). The authors interpreted the increase in susceptibility as a consequence of increased iron content, but noted that demyelination may play an additive role. In a small clinical imaging study, we demonstrated that patients with active RRMS contained significantly more lesions with high susceptibility on phase imaging than patients with chronic, stable disease (27). Furthermore, we found in a retrospective study, where susceptibility was quantified in white matter lesions of different ages, tissue susceptibility was isointense in Gd-enhancing lesions, and increased rapidly after enhancement subsided, suggesting that lesions acquired iron as they transitioned from an acute to a chronic active state. The elevated susceptibility was stable for approximately 4 years and then decayed to levels similar to that of NAWM (Figure 1) (111). This time course of tissue susceptibility was recently confirmed in a separate longitudinal study with MS patients (112). On a cellular level, the isointense susceptibility in enhancing lesions may be explained by the reduced capacity of myelin-phagocytosing macrophages to take up iron (27), consistent with the M2-like phenotype of myelin-laden macrophages (26). As the lesion evolves, myelin-laden macrophages continue to break down ingested myelin and eventually exit the lesion center. Activated non- or slowly phagocytosing myeloid cells at the lesion rim accumulate iron and adopt a chronic inflammatory state (75) that may persist for several years (111, 112).

In a recent prospective imaging study using phase imaging, persistence of phase rims in white matter lesions was associated with increased lesion T1 hypointensities, a marker for tissue damage (113). In addition, Dal-Bianco et al. reported that white matter lesions with phase-positive rims slowly expanded over time, supporting the idea that iron-positive microglia/macrophages are associated with chronic, slow inflammatory demyelination (24). It is tempting to speculate that high prevalence of lesions with hyperintense rims is associated with a more severe disease course and/or disease progression; however, these data are not yet available. Prospective studies examining these correlations are currently ongoing at our centers.

The prevalence of white matter lesions with hyperintense rims on QSM and phase imaging varies widely, ranging from 0 (113) to 32% (114). This variability is unsurprising given the different imaging techniques, resolutions, and patient cohorts used in these studies. An imaging study on MS patients from our group revealed that 21% of lesions visible on QSM had a hyperintense rim, and 79% displayed homogenous or heterogenous distribution patterns (115). Our preliminary data from a combined imaging and histology study of MS brain tissue suggest that heterogenous QSM patterns were typically associated with the presence of heme within enlarged blood vessels in MS lesions. We have currently no data to explain homogenously increased susceptibility throughout lesions, but hypothesize that absence of myelin drives the susceptibility increase in these lesions.

In summary, although susceptibility weighted imaging cannot distinguish between iron accumulation and myelin loss, increased susceptibility at the lesion rim likely represents chronically activated, iron-positive microglia and macrophages. Moreover, longitudinal imaging studies of MS patients using QSM suggest that iron-positive lesions persist for many years and are associated with increased tissue loss and slow expansion (24, 113).

## Clinical Implications

Based on the above studies, high tissue susceptibility in white-matter lesions may be useful as a biomarker for chronic active lesions. Although the detrimental effect of smoldering, low-grade inflammation on the surrounding parenchyma has been demonstrated (24, 113), it is unknown if the presence of hyperintense susceptibility rim lesions predict a more severe clinical course; studies are ongoing that examine this association. Moreover, we are testing the ability of current MS treatments to remove iron from existing white matter lesions in MS patients. Of particular interest are MS medications that penetrate the BBB and act directly on microglia, such as dimethyl fumarate (Tecfidera™), fingolimod (Gilenya™), and Laquinimod (116–118).

Since QSM can be rapidly and reliably acquired with standard field strength (3T) MRI scanners, it can easily be implemented in clinical settings and broadly used for MS patient care. Thus, iron-sensitive imaging may become an important imaging modality to detect chronic inflammation in MS patients that appear stable on conventional MRI but have a high burden of lesional microglial activation.

## Summary and Outlook

We reviewed iron metabolism in macrophages/microglia, iron accumulation in MS lesions, and iron-sensitive imaging studies in MS tissue and patients. Iron is taken up by M1-polarized macrophages/microglia, which may further increase their pro-inflammatory properties. Iron can be visualized with MR sequences sensitive to tissue susceptibility. In MS patients, high susceptibility in white matter lesions can persist for several years after lesion formation, suggesting that iron-positive myeloid cells are present in MS lesions for prolonged periods of time. In addition, high susceptibility is associated with increase tissue loss and lesion expansion.

Therefore, the emerging picture suggests that iron-positive microglia and macrophages in chronic active MS lesions constitute a distinct, previously unappreciated inflammatory compartment that may be a significant contributor to tissue damage, disease severity, and/or progression. Reducing chronic inflammation associated with iron deposition in MS lesions with existing or novel MS therapies may be of high benefit to patients.

## Author Contributions

KG and DP wrote the manuscript. MM, KG, and TDN conducted experiments. All authors contributed to analysis and interpretation.

## Conflict of Interest Statement

The authors declare that the research was conducted in the absence of any commercial or financial relationships that could be construed as a potential conflict of interest.
